# Coexistence of *tmexCD-toprJ*, *bla*_NDM-1_, and *bla*_IMP-4_ in One Plasmid Carried by Clinical *Klebsiella* spp.

**DOI:** 10.1128/spectrum.00549-22

**Published:** 2022-06-01

**Authors:** Tao Xiao, Kai Peng, Qi Chen, Xueqin Hou, Weifeng Huang, Hong Lv, Xiaorong Yang, Gaopeng Lei, Ruichao Li

**Affiliations:** a Center for Disease Control and Prevention of Sichuan Province, Chengdu, Sichuan Province, People’s Republic of China; b Jiangsu Co-Innovation Center for Prevention and Control of Important Animal Infectious Diseases and Zoonoses, College of Veterinary Medicine, Yangzhou University, Yangzhou, Jiangsu Province, People’s Republic of China; c Institute of Comparative Medicine, Yangzhou University, Yangzhou, Jiangsu Province, People’s Republic of China; d Suining Center for Disease Control and Prevention, Suining, Sichuan Province, People’s Republic of China; e Guangyuan Center for Disease Control and Prevention, Guangyuan, Sichuan Province, People’s Republic of China; University Paris-Saclay, AP-HP Hôpital Antoine Béclère, Service de Microbiologie, Institute for Integrative Biology of the Cell (I2BC), CEA, CNRS

**Keywords:** *Klebsiella* spp., carbapenem resistance, coexistence, plasmid, tigecycline resistance

## Abstract

In clinical practice, carbapenems and tigecycline are considered significant options for treating infections caused by multidrug-resistant *Klebsiella* spp. The continual evolution of resistance mechanisms to carbapenems and tigecycline is shattering the present condition. Meanwhile, convergence of the two resistance mechanisms in a single strain has been reported repeatedly, posing a significant threat to public health and safety. In this study, two carbapenem- and tigecycline-resistant *Klebsiella* species were obtained from patients and investigated using antimicrobial susceptibility testing, conjugation assay, whole-genome sequencing, and bioinformatics analysis. In Klebsiella variicola FK2020ZBJ35, an untransferable multidrug IncFIB(Mar)/IncHI1B-like plasmid carrying *tmexCD2-toprJ2*, *bla*_IMP-4_, and *bla*_NDM-1_ was discovered, as was a similar plasmid carrying *tmexCD1-toprJ1* and *bla*_IMP-4_ in Klebsiella quasipneumoniae 2019SCSN059. Genetic context analysis found that two distinct *tmexCD-toprJ* variants were detected in comparable mobile units with genetic array *int-int-hp-hp-tnfxB-tmexCD-toprJ* and integrated into separate genetic locations. *bla*_IMP-4_ and *bla*_NDM-1_ were carried by an integron In*1377* and a truncated Tn*3000*, respectively. These findings revealed that the carbapenem and tigecycline resistance genes carried by the two strains were located on mobile elements and might potentially transmit horizontally to additional strains. Furthermore, our findings showed that IncFIB(Mar)/IncHI1B-like plasmids represent a significant reservoir of essential resistance genes that warrants continued monitoring.

**IMPORTANCE** Tigecycline is an essential antibiotic that is used to treat infections caused by carbapenem-resistant Klebsiella pneumoniae (CRKP). The emergence of high-level tigecycline-resistant CRKP poses a serious hazard to human health. This work screened two tigecycline-resistant CRKP strains from clinical patients and found a type of plasmid that encoded carbapenemase and TmexCD-ToprJ in *Klebsiella*. Importantly, one plasmid cocarried *tmexCD-toprJ*, *bla*_NDM-1_, and *bla*_IMP-4_, hinting that this plasmid could be a critical vector for superbug development. Furthermore, we discovered that the carbapenem and tigecycline resistance genes are located in mobile units by genetic structure analysis. Our research tracks the formation of clinically super-resistant Gram-negative bacteria.

## INTRODUCTION

*Klebsiella* is a genus of diverse bacterial pathogens that originate in the gut flora and is a common cause of nosocomial infections such as pneumonia, septicemia, and urinary tract infections (1). Up to now, many species and subspecies of the genus *Klebsiella* have been identified and consist of Klebsiella pneumoniae, Klebsiella quasipneumoniae, Klebsiella granulomatis, Klebsiella michiganensis, Klebsiella oxytoca, Klebsiella variicola, and so on (2, 3). Recently, K. pneumoniae has been classified into three distinct species as follows: K. pneumoniae (KPI), Klebsiella quasipneumoniae (KpII), and Klebsiella variicola (KpIII) according to a genome-wide analysis (4). Among them, K. pneumoniae has the most widespread distribution and is a common opportunistic pathogen capable of causing a wide range of community-acquired and nosocomial infections (5). By contrast, the other two phylogroups, *K. quasipneumoniae* and *K. variicola*, have been less reported as causing infections in humans (5). Because it is difficult to differentiate the phylogroups of K. pneumoniae using regular molecular typing methods in clinical laboratories, researchers have underestimated their true prevalence (6). The difference between various species of bacteria is becoming increasingly obvious with the advancement of whole-genome sequencing. Infections produced by *K. quasipneumoniae* and *K. variicola* are becoming more prevalent in hospitals, making them emergent and common pathogens in humans (7–9).

Carbapenems are a class of atypical beta-lactam antibiotics with broad-spectrum high antibacterial activity, which are commonly used in the treatment of infections caused by multidrug-resistant Gram-negative bacteria (10). Tigecycline is a glycylcycline antibiotic and is added as a last-resort therapeutic option for patients infected with multidrug-resistant bacteria (11). In clinical practice, carbapenems and tigecycline are significant antibiotics for treating multidrug-resistant *Klebsiella*. Many carbapenemase-encoding genes have emerged in *Klebsiella*, posing a significant barrier to clinical infection management (12, 13). More significantly, in K. pneumoniae, a new plasmid-mediated resistance-nodulation-division (RND)-type efflux pump gene cluster, *tmexCD1-toprJ1*, was recently found, further reducing tigecycline sensitivity (14). Meanwhile, convergence of carbapenemase encoding genes and TmexCD-ToprJ in one plasmid has been reported (15). The spread of such plasmids in various bacteria may mean that there will be limited therapy options for bacterial illnesses tomorrow. In this study, we screened *tmexCD-toprJ*-positive *Klebsiella* in carbapenem-resistant clinical *Klebsiella* and identified two *Klebsiella* strains that encode carbapenemase and TmexCD-ToprJ.

## RESULTS

### Characteristic of the two *tmexCD-toprJ*-positive carbapenem-resistant strains.

Two *tmexCD-toprJ*-positive isolates, 2019SCSN059 and FK2020ZBJ35, were identified from 165 carbapenem-resistant clinical *Klebsiella* spp. in Sichuan, China, in 2019. Using matrix-assisted laser desorption ionization–time of flight mass spectrometry (MALDI-TOF MS), the two resistant isolates were identified as K. pneumoniae, and ribosomal multilocus sequence typing (rMLST) validated them as K. quasipneumoniae and K. variicola (https://pubmlst.org/species-id). Isolate 2019SCSN059 belonged to ST2421-1LV/KL107-like and isolate FK2020ZBJ35 belonged to ST697/KL103 according to MLST and *in silico* serotyping analyses. Antimicrobial susceptibility tests revealed that the two isolates were resistant to ampicillin, ceftazidime, ampicillin/sulbactam, imipenem, tetracycline, tigecycline, cefoxitin, cefazolin, ciprofloxacin, meropenem, and amikacin and had a high MIC for azithromycin but were sensitive to colistin (see Table S1 in the supplemental material). Isolate 2019SCSN059 was also resistant to gentamicin and trimethoprim-sulfamethoxazole. Apart from colistin, FK2020ZBJ35 was sensitive to gentamicin and trimethoprim-sulfamethoxazole (see Table S1).

### Genome feature of the two strains.

We got the draft genomes of the two strains after *de novo* assembly using short-read data. Isolate 2019SCSN059 was assembled into 181 contigs with an average GC content of 57%. Isolate FK2020ZBJ35 was assembled into 54 contigs with an average GC content of 56.8%. Resistance gene analysis showed that both of them harbored many antibiotic resistance genes (ARGs) (Table 1). Significantly, the tigecycline resistance gene cluster *tmexCD-toprJ* and carbapenem resistance gene *bla*_IMP-4_ were simultaneously detected in the two strains. In addition, two different variants of gene cluster *tmexCD-toprJ* were detected in the two strains. Strain 2019SCSN059 carried *tmexCD1-toprJ1* and strain FK2020ZBJ35 carried *tmexCD2-toprJ2*. Apart from *tmexCD2-toprJ2* and *bla*_IMP-4_, FK2020ZBJ35 also carried another carbapenem resistance gene, *bla*_NDM-1_. In addition to ARGs, we also analyzed virulence-associated genes and insertion sequences (ISs) of the two strains. None of the acquired virulence genes were detected in them. However, we detected many ISs in the genomes of the two strains (see Table S2 in the supplemental material).

**TABLE 1:** Basic information of *tmexCD-toprJ*-bearing plasmids investigated in this study

Strain	Sequence type	Species	Assembly method	Sequencing platform	Location of *tmexCD-toprJ*	*tmexCD-toprJ*-harboring plasmid replicons	*tmexCD-toprJ* variant	Resistance genes
2019SCSN059	ST2421-1LV	Klebsiella quasipneumoniae	Unicycler	MinION, Illumina	p2019SCSN059_tmexCD_333k (333,095 bp)	IncFIB(Mar)-like, IncHI1B-like	*tmexCD1-toprJ1*	*tet*(A), *tmexC1-tmexD1-toprJ1*, *bla*_IMP-4_, *strB*, *strA*, *aac*(3)*-lld*, *bla*_TEM-1B_, *aac*(*6’*)*-lb-cr*, *bla*_OXA-1_, *catB*, *sul1*, *bla*_PER-1_, *aadA5*
FK2020ZBJ35	ST697	Klebsiella variicola	Unicycler	MinION, Illumina	pFK2020ZBJ35_tmexCD_325k (325,393 bp)	IncFIB(Mar)-like, IncHI1B-like, IncU	*tmexCD2-toprJ2*	*tmexC2-tmexD2-toprJ2*, *qnrS1*, *bla*_NDM-1_, *strA*, *bla*_SFO-1_, *aac*(*6’*)*-lld*, *bla*_IMP-4_

**FIG 1:**
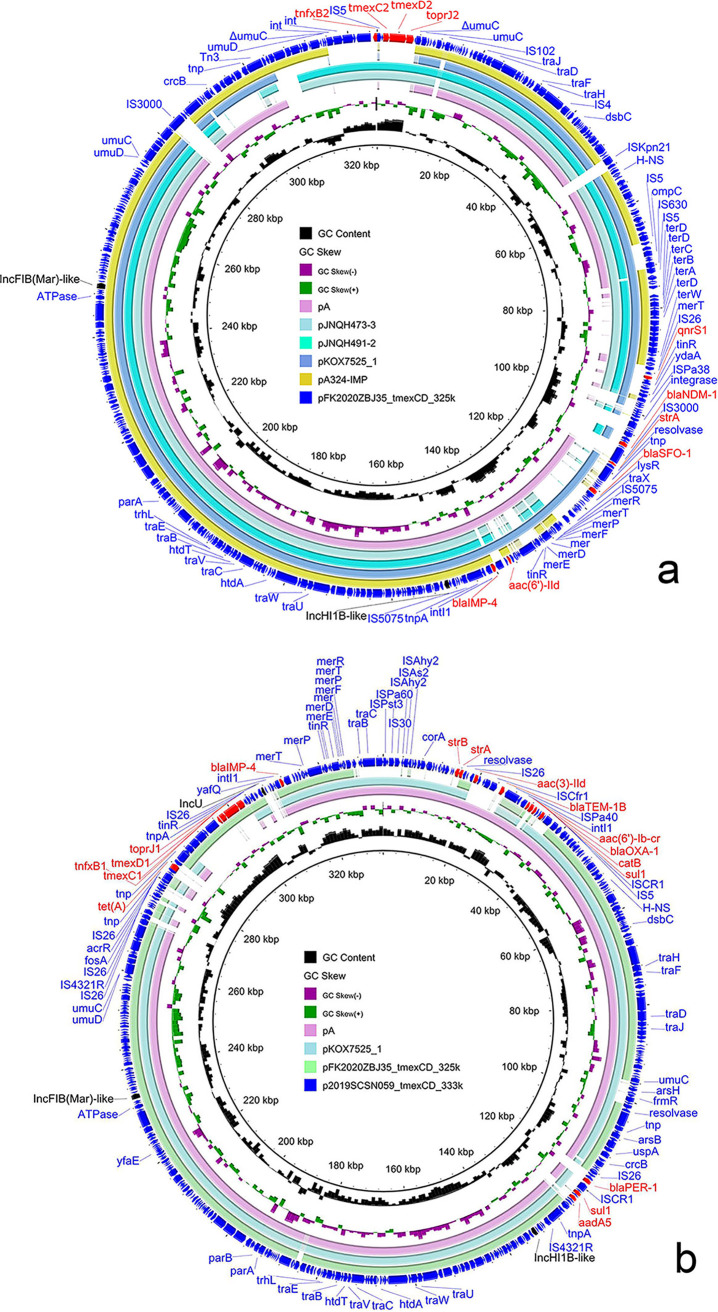
Structure analysis of *tmexCD-toprJ*-bearing plasmids. Plasmid pFK2020ZBJ35_tmexCD_325k and plasmid p2019SCSN059_tmexCD_333k were used as reference plasmids in panels a and b, respectively. The annotations of plasmid replicon genes were black and resistance genes were red.

Many plasmid replicon genes were detected in the draft genomes of the two strains, indicating that ARGs might be transmitted via plasmids. In order to investigate the locations of ARGs, the genomic DNA of the two strains were subjected to nanopore long-read sequencing. Although we were unable to obtain complete genome sequences of the two strains utilizing a hybrid assembly strategy combining short-read and long-read data, we were able to effectively obtain a portion of the multiple resistance complete plasmids that they have. In 2019SCSN059, a 333-kb plasmid called p2019SCSN059_tmexCD_333k was successfully built as was a 325-kb plasmid named pFK2020ZBJ35_tmexCD_325k in FK2020ZBJ35. The two plasmids contained the majority of ARGs, containing *tmexCD-toprJ*, *bla*_IMP-4_, and *bla*_NDM-1_ (Fig. 1).

### Transmissibility of *tmexCD-toprJ*, *bla*_IMP-4_, and *bla*_NDM-1_.

The conjugation assay was originally carried out with Escherichia coli C600 as the recipient. Despite several tries, we were unable to recover transconjugants. We assumed that plasmids from *Klebsiella* spp. are host specific and cannot be transferred to E. coli. Then, we used hygromycin-resistant sequence type 11 (ST11) K. pneumoniae HS11286YZ6 as the recipient strain for the conjugation experiment to remove the host range effect on plasmid transfer. However, we failed once more. The findings revealed that such plasmids could not be transmitted in a laboratory setting using a conjugation experiment.

### Genetic features of the two plasmids coharboring carbapenems and tigecycline resistance genes.

Three replicon genes, IncFIB(Mar)-like, IncHI1B-like, and IncU, were discovered in p2019SCSN059_tmexCD_333k, while two replicon genes, IncFIB(Mar)-like and IncHI1B-like, were found in pFK2020ZBJ35_tmexCD_325k. The genes *tmexCD-toprJ* and *bla*_IMP-4_ were carried by each of them. Furthermore, *bla*_NDM-1_ was carried by pFK2020ZBJ35_tmexCD_325k (Fig. 1). To the best of our knowledge, this is the first report of a plasmid coharboring *tmexCD1-toprJ1*, *bla*_NDM-1_, and *bla*_IMP-4_. Furthermore, numerous other resistance genes integrated into p2019SCSN059_tmexCD_333k and pFK2020ZBJ35_tmexCD_325k, making them multiple resistance plasmids. The backbones of the two plasmids shared 83% sequence coverage and 99.9% nucleotide identify. In addition, numerous homologous plasmids were found in the NCBI nr database using the BLASTn program (Fig. 1). Some of the homologous plasmids in the NCBI nr database carried one or two of *tmexCD-toprJ*, *bla*_NDM-1_, and *bla*_IMP-4_. Hence, we assumed that such plasmids might be a potential transmission vector for *tmexCD-toprJ*, *bla*_NDM-1_, and *bla*_IMP-4_. Meanwhile, BLASTn results revealed that practically all of these plasmids were discovered in *Klebsiella* spp., indicating that they may have originated in *Klebsiella* spp. As bacteria acquire such plasmids, they will acquire the resistance phenotypes encoded by the plasmids. As a result, monitoring the horizontal spread of such plasmids in *Klebsiella* spp. or other bacteria species is still necessary.

### Genetic contexts of *tmexCD-toprJ*, *bla*_IMP-4_, and *bla*_NDM-1_.

**FIG 2:**
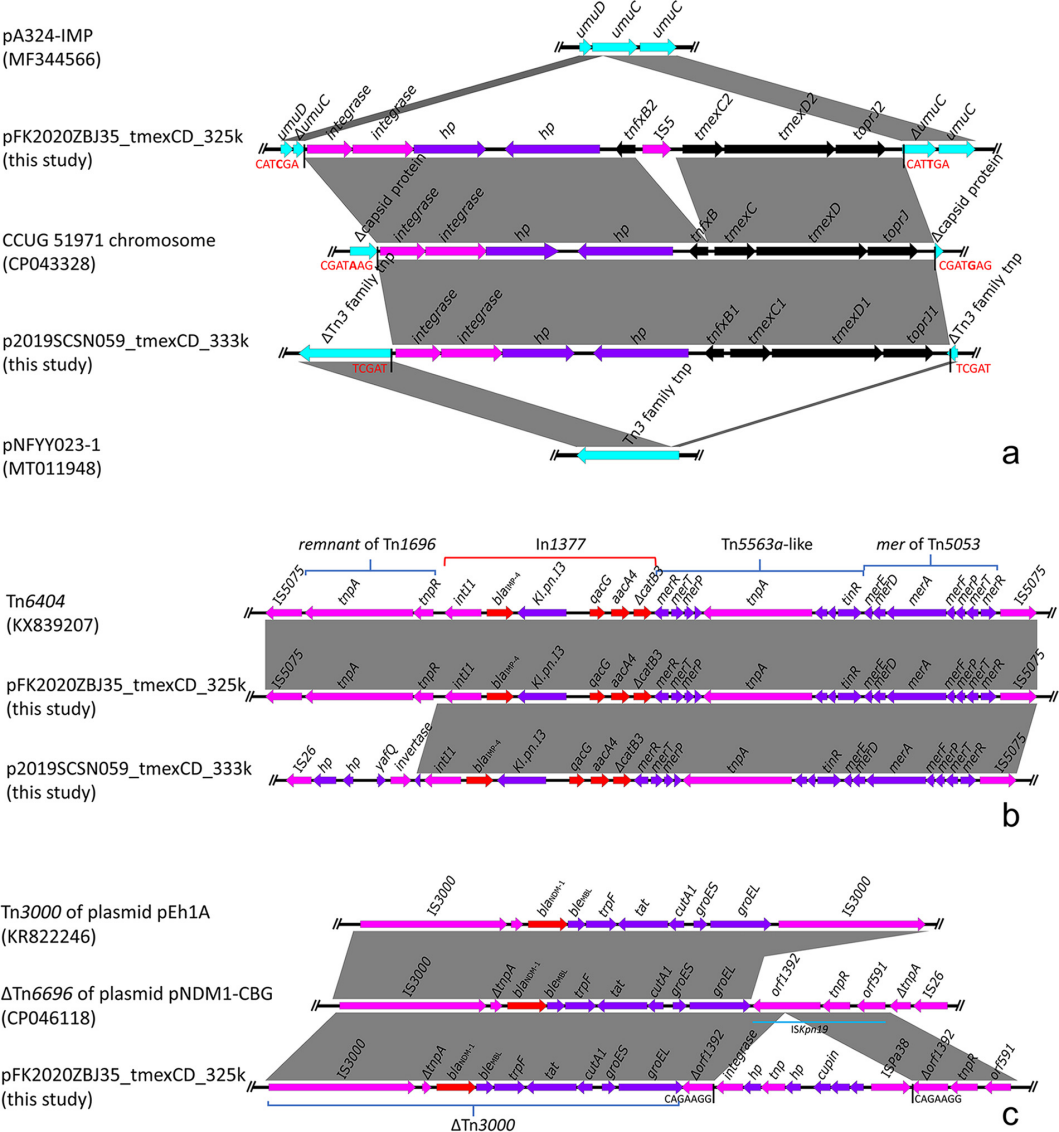
Colinear analyses for the genetic context of *tmexCD-toprJ*, *bla*_IMP-4_, and *bla*_NDM-1_. (a) The genetic environment of *tmexCD-toprJ*. Direct repeat sequences were highlighted by red fonts. Intact *umuD-umuC-umuC* and Tn*3* structure before insertions of *tmexCD-toprJ*-containing regions were found in pA342-IMP and pNFYY023-1. (b) The genetic structure of *bla*_IMP-4_. A complete Tn*6404* was detected in plasmid pFK2020ZBJ35_tmexCD_325k. (c) The genetic environment of *bla*_NDM-1_. The gene *bla*_NDM-1_ was found in an interrupted Tn*3000*.

To further analyze the genetic contexts of *tmexCD-toprJ*, *bla*_IMP-4_, and *bla*_NDM-1_, the structures of the two plasmids were investigated in detail. Gene cluster *tmexCD1-toprJ1* in plasmid p2019SCSN059_tmexCD_333k was inserted into a Δ Tn*3* family transposase, which created 5-bp (TCGAT) direct repeats. In plasmid pFK2020ZBJ35_tmexCD_325k, *tmexCD2-toprJ2* was inserted into a *umuC* gene and created 6-bp (CATCGA/CATTGA) direct repeats. Both insertion regions contain two *int* genes, two hypothetical protein-encoding genes, and a *tmexCD-toprJ* gene cluster. Importantly, we observed direct repeats around the insertion regions, which is a typical phenomenon of the transposition process (Fig. 2a). Apart from the two strains, we also found the *tmexCD-toprJ* insertion event in other strains, for example, in the chromosome of strain CCUG (Fig. 2a). In addition, we also found the intact gene *umuC* and Tn*3* family transposase before they were interrupted in some plasmids (Fig. 2a). These phenomena further demonstrated the high mobilization of the *tmexCD-toprJ-*containing insertion regions.

The carbapenemase-encoding gene *bla*_IMP-4_ was found in both plasmids p2019SCSN059_tmexCD_333k and pFK2020ZBJ35_tmexCD_325k. Genetic structure analysis showed that *bla*_IMP-4_ was located in a class 1 integron named In*1377* in the two plasmids (Fig. 2b). The integron In*1377* also carries *qacG*, *aacA4*, and Δ *catB3* in addition to *bla*_IMP-4_. In plasmid pFK2020ZBJ35_tmexCD_325k, In*1377* was carried by a complex transposon Tn*6404*, demonstrating that *bla*_IMP-4_ was highly mobilizable (Fig. 2b). Tn*6404* carrying In*1377* was also discovered in plasmid p2019SCSN059_tmexCD_333k. However, the genes IS*5075*, *tnpA*, and *tnpR* in Tn*6404* of p2019SCSN059_tmexCD_333k were deleted and replaced by IS*26*-*hp*-*hp*-*yafQ*-*invertase* (Fig. 2b). The structural alteration of Tn*6404* might prevent it from spreading further.

Apart from *tmexCD-toprJ* and *bla*_IMP-4_, plasmid pFK2020ZBJ35_tmexCD_325k also carried a *bla*_NDM-1_ gene. According to genetic context analysis, *bla*_NDM-1_ was integrated into a Δ Tn*3000* transposon, which lost a downstream IS*3000* (Fig. 2c). This seems to be a recurrent occurrence in the genetic structure of *bla*_NDM-1_. The downstream IS*3000* was replaced by an IS*Kpn19* in both plasmids pNDM1-CBG and pFK2020ZBJ35_tmexCD_325k. Meanwhile, a genetic structure, *int*-*hp*-*tnp*-*hp*-*cupin*-*hp*-*hp*-IS*Pa38*, disrupted the IS*Kpn19* of pFK2020ZBJ35_tmexCD_325k.

## DISCUSSION

In recent years, carbapenem-resistant Klebsiella pneumoniae (CRKP) has become more common in nosocomial infections, posing a significant barrier to clinical treatment (12). The acquisition of carbapenemase genes, which produce enzymes capable of hydrolyzing carbapenems, is the principal source of carbapenem resistance in K. pneumoniae (16). Most carbapenemase genes are carried by plasmids, which are extrachromosomal DNA elements that may self-replicate and move horizontally, substantially facilitating their transmission in bacteria (17, 18). Hence, carbapenem-resistant K. pneumoniae has emerged as a global threat. In view of this, tigecycline has been utilized as one of the “last resort” antibiotics in the treatment of CRKP infection (19, 20). However, the emergence of plasmid-encoding *tet*(X) and *tmexCD1-toprJ1* weakened the function of tigecycline (14, 21). Even worse, the coexistence of carbapenemase genes with *tet*(X) or *tmexCD1-toprJ1* in *Klebsiella* spp. has been reported (15, 22). This highlighted that when patients are infected with such *Klebsiella* spp., there is a limited choice for treatment. In this investigation, we screened 165 clinical carbapenem-resistant *Klebsiella* isolates for *tet*(X) and *tmexCD-toprJ*-positive bacteria and found one *K. quasipneumoniae* isolate cocarrying *tmexCD1-toprJ1* and *bla*_IMP-4_ and one *K. variicola* isolate coharboring *tmexCD2-toprJ2*, *bla*_IMP-4_, and *bla*_NDM-1_. Mobile tigecycline resistance genes were found in a small percentage of clinical carbapenem-resistant *Klebsiella* isolates. Due to the increased use of tigecycline in clinical settings, tigecycline- and carbapenem-resistant *Klebsiella* isolates may pose a greater threat to human health. As a result, we should pay more attention to control the generation and propagation of CRKP.

In this study, we found that the *tmexCD-toprJ* and carbapenemase genes were coded by the same plasmids (plasmid p2019SCSN059_tmexCD_333k in strain 2019SCSN059 and plasmid pFK2020ZBJ35_tmexCD_325k in strain FK2020ZBJ35) in both strains. Interestingly, p2019SCSN059_tmexCD_333k and pFK2020ZBJ35_tmexCD_325k shared a similar plasmid backbone. This suggested that such plasmids were adapted to *Klebsiella* spp. and may have evolved for a long period in *Klebsiella* spp. Under laboratory circumstances, the two plasmids were unable to be transmitted into recipients via conjugation. Even so, we cannot ignore the natural behavior of these high-risk plasmids. Furthermore, we discovered a number of comparable plasmids in the NCBI nr database using the BLASTn program. Some of them carried both *tmexCD-toprJ* and *bla*_NDM-1_ and had been reported in a previous study (15). Some of them carried only *tmexCD-toprJ* or carbapenemase genes (Fig. 2a). The findings suggested that such plasmids may serve as a reservoir for a large number of resistance genes. We detected *tmexCD2-toprJ2*, *bla*_IMP-4_, and *bla*_NDM-1_ in pFK2020ZBJ35_tmexCD_325k simultaneously for the first time, which further presented that various resistance genes, including carbapenemase genes, could integrate into such plasmids, thereby making them multidrug-resistant plasmids. Plasmid replicon analysis showed that these plasmids harbored two conserved replicon genes. Here, we call them IncFIB(Mar)-like and IncHI1B-like, which were most homologous to IncFIB(Mar) and IncHI1B. Although prior research identified these plasmids as IncHI1B plasmids (15), we advise that additional genetic analysis and detailed classification be performed in future studies.

Genetic context analysis revealed that *tmexCD-toprJ* was positioned in a conserved mobile unit with genetic arrangement *int*-*int*-*hp*-*hp*-*tnfxB*-*tmexCD*-*toprJ* in the two plasmids. Previous studies have uncovered that similar mobile units containing *tmexCD-toprJ* were present in many strains (23, 24). As yet, no studies have been conducted to demonstrate the mobility such mobile units. However, according to genetic analysis, we were able to discover several pieces of evidence of mobile unit transfer. Interestingly, different variants of *tmexCD-toprJ* shared a similar genetic structure (14, 15, 25), implying that these *tmexCD-toprJ* variants originated from a common ancestor and then propagated to different bacteria. It should be noted that IS*26*-mediated movement of *tmexCD-toprJ* has been detected in many strains (24, 26), and we should therefore present an effective way to prevent further dissemination of *tmexCD-toprJ*. Apart from *tmexCD-toprJ*, *bla*_IMP-4_ and *bla*_NDM-1_ were also located in mobile elements. The *bla*_IMP-4_ was carried by a class I integron in the two plasmids, which showed 100% identity with In*809*-like integron in Tn*6404* of plasmid pKP1814-1 (27). Afterward, the In*809*-like was observed in another plasmid, pA708-IMP, and was renamed In*1377* (28). The emergence of In*1377* continuously in different plasmids indicated that class 1 integrons played a significant role in propagating *bla*_IMP-4_. Unlike *bla*_IMP-4_, *bla*_NDM-1_ was found in a Δ Tn*3000* transposon in plasmid pFK2020ZBJ35_tmexCD_325k. Tn*3000* has been observed in many different types of plasmids from different bacteria for many years (29), and it was an essential vector for the horizontal transmission of *bla*_NDM-1_. To date, many genetic structures of *bla*_NDM-1_ have been linked to Tn*3000* (30), including the genetic context of *bla*_NDM-1_ that we discovered. We should pay more attention to the genetic structure of *bla*_NDM-1_ derived from Tn*3000* to understand its possible transmission mechanisms. In conclusion, the acquisition of multiple mobilizable resistance genes is the primary cause of the creation of multidrug-resistant plasmids. To curb the spread of ARGs, it is critical to understand their genetic background.

## MATERIALS AND METHODS

### Bacterial isolates.

In 2019, a total of 165 carbapenem-resistant *Klebsiella* isolates were collected from different hospitals in Sichuan, China. These isolates were sent to the Center for Disease Control and Prevention of Sichuan Province, and whole-genome sequencing was performed. The tigecycline resistance genes *tet*(X) and *tmexCD-toprJ* were then screened in the 165 genomes. There were two *tmexCD-toprJ*-positive isolates discovered. Strain 2019SCSN059 was isolated from a sputum sample and strain FK2020ZBJ35 isolated from a secretion sample. Strains were grown at 37°C on Luria-Bertani (LB) plates and identified as K. pneumoniae through matrix-assisted laser desorption ionization–time of flight mass spectrometry (MALDI-TOF MS) (Bruker, Bremen, Germany). Prior to following experiments, two isolates were kept at −80°C in LB broth containing 20% glycerol.

### Antimicrobial susceptibility testing and conjugation assay.

We used broth microdilution to determine the MICs of the two carbapenem- and tigecycline-resistant bacteria as per Clinical and Laboratory Standards Institute (CLSI) standards. E. coli ATCC25922 was used for quality control. The resistance breakpoints for tigecycline, colistin, and cefazolin were interpreted according to the European Committee on Antimicrobial Susceptibility Testing (EUCAST) (http://www.eucast.org/clinical_breakpoints/), due to data deficiency in CLSI. E. coli C600 (Rif^r^) and *K. pneumonaie* HS11286YZ6 (Hm^r^) were used as recipients in the conjugation studies. The donor and recipient strains were grown in LB broth until they reached the logarithmic growth phase and then combined 1:1 and cultured overnight on LB agar plates. The transconjugants were next screened on LB agar plates with rifampin (300 mg/L) or hygromycin (300 mg/L) as well as tigecycline (2 mg/L) and validated using PCR targeting the *tmexCD-toprJ* gene cluster (31). In detail, for K. pneumoniae HS11286YZ6, we verified the transconjugant as positive when it was K. pneumoniae and shared the same ST type with HS11286YZ6. For E. coli C600, the positive transconjugants were identified as E. coli and positive for *tmexCD-toprJ*.

### Genomic DNA extraction and sequencing.

In order to decipher the genetic structure features of the two carbapenem and tigecycline resistant strains. We extracted genomic DNA using FastPure bacteria DNA isolation minikit (Vazyme, China) following the instructions of the manufacturer. The purified genomic DNA was quantified spectrophotometrically (Titertek-Berthold Colibri, Berthold, Germany). Then, the genomic DNA was sequenced combining short- and long-read sequencing methods. The short-read sequencing (2 × 150bp) was performed using the Illumina HiSeq 2500 platform. The long-read sequencing was conducted using the MinION platform developed by Oxford Nanopore Technologies. The long-read sequencing library was prepared using the SQK-LSK109 1D ligation genomic DNA kit according to the user handbook. MinION sequencing was then carried out with R9.4 flow cells and managed with MinKNOW.

### Data analysis.

The short-read raw reads were filtered to remove low-quality base and adapters using Trimmomatic (v 0.39) (32). Then, clean short-read data and long-read data were *de novo* assembled using SPAdes (v 3.1.13) (33) and Flye (v 2.8-b1674) (34), respectively. Meanwhile, to get complete bacterial genomes, a hybrid assembly technique integrating long-read and short-read sequencing data was used (35). Functional annotation of the assembled bacterial genomes was performed by a web-based RAST annotation engine (36, 37). Antibiotic resistance genes (ARGs) and plasmid replicon types in bacterial genomes were identified using the abricate tool (https://github.com/tseemann/abricate) based on the NCBI AMRFinderPlus (38) and PlasmidFinder (39) databases. Insertion sequences (ISs) was detected using an online tool ISFinder (40). Multilocus sequence typing (MLST) of complete bacterial genomes were performed using the mlst tool (https://github.com/tseemann/mlst). The virulence factors and serotype were identified using the kleborate (41) software. Plasmid comparisons and genetic context comparisons were visualized using the BRIG (42) and Easyfig (43) tools, respectively.

### Data availability.

The draft genome sequences in this study were deposited into the National Center for Biotechnology Information under BioProject PRJNA798532. The complete plasmid sequences of p2019SCSN059_tmexCD_333k and pFK2020ZBJ35_tmexCD_325k were submitted to the NCBI database with accession numbers ON169978 and ON169979.
