# Supplementary material for: Coexistence of tmexCD-toprJ, blaNDM-1, and blaIMP-4 in One Plasmid Carried by Clinical Klebsiella spp
Source: Microbiol Spectr. 2022 Jun 1;10(3):e00549-22. doi: 10.1128/spectrum.00549-22 (PMC9241619; doi:10.1128/spectrum.00549-22)
Supplement: Supplemental Material — Tables S1 and S2. Download spectrum.00549-22-s0001.pdf, PDF file, 0.1 MB [file spectrum.00549-22-s0001.pdf]

Supplementary Table 1. MIC[a] values (mg/L) of different antimicrobials among strains investigated in this study.

Isolates	antibiotics[b]														
/	AMP	CAZ	AMS	IMP	TET	TIG	CL	CFX	CFZ	GEN	SXT	AZM	CIP	MEM	AMK
Resistance breakpoints	≥32	≥16	≥32/16	≥4	≥16	>0.5	>2	≥32	>4	≥16	≥4/76	/	≥1	≥4	≥64
2019SCSN059	>64	>32	>64/32	4	32	32	≤0.25	>64	32	32	>8/152	>64	16	4	>64
FK2020ZBJ035	>64	>32	>64/32	32	16	8	≤0.25	>64	32	≤0.5	≤0.25/0.475	>64	1	64	>64
ATCC25922	4	≤0.5	4/2	≤0.25	≤0.5	≤0.5	≤0.25	4	2	≤0.5	≤0.25/0.475	≤0.5	1	≤0.5	2

[a] MIC minimum inhibitory concentration.

[b] AMP ampicillin, CAZ ceftazidime, AMS ampicillin/sulbactam, IMP imipenem, TET tetracycline, TIG tigecycline, CL colistin, CFX cefoxitin, CFZ cefazolin, GEN gentamicin, SXT trimethoprim-sulfomethoxazole, AZM azithromycin, CIP ciprofloxacin, MEM meropenem, AMK amikacin.

Supplementary Table 2. Distribution of insertion sequences in strain 2019SCSN059 and FK2020ZBJ035.

Isolates	Contigs	Genes	Coverage(%)	Identity(%)
2019SCSN059	NODE_107_length_1381_cov_70.228528	IS*1006*	100	99.88
2019SCSN059	NODE_112_length_1315_cov_278.667205	IS*SoEn2*	100	86.69
2019SCSN059	NODE_116_length_1185_cov_498.757220	IS*5075*	89	99.75
2019SCSN059	NODE_120_length_1067_cov_206.261616	IS*Vsa3*	86.49	100
2019SCSN059	NODE_127_length_859_cov_224.500000	IS*102*	80.98	94.05
2019SCSN059	NODE_128_length_802_cov_1691.655172	IS*26*	97.8	100
2019SCSN059	NODE_129_length_767_cov_201.066667	IS*1N*	100	99.61
2019SCSN059	NODE_28_length_74856_cov_127.734712	IS*4*	100	88.85
2019SCSN059	NODE_28_length_74856_cov_127.734712	IS*1618*	100	90.18
2019SCSN059	NODE_43_length_31756_cov_91.293728	IS*1X4*	100	97
2019SCSN059	NODE_48_length_23742_cov_159.360025	IS*Pst3*	99.96	97.97
2019SCSN059	NODE_48_length_23742_cov_159.360025	IS*As2*	100	99.45
2019SCSN059	NODE_54_length_18075_cov_92.995722	IS*Ec21*	100	92.58
2019SCSN059	NODE_57_length_15149_cov_92.130706	IS*1541A*	100	88.72
2019SCSN059	NODE_65_length_7360_cov_452.774406	IS*5*	100	99.5
2019SCSN059	NODE_65_length_7360_cov_452.774406	IS*Ec33*	100	99.65
2019SCSN059	NODE_65_length_7360_cov_452.774406	IS*5*	100	99.5
2019SCSN059	NODE_71_length_5652_cov_169.040179	IS*4321R*	100	99.62
2019SCSN059	NODE_72_length_5315_cov_159.782551	IS*6100*	100	100
2019SCSN059	NODE_7_length_207361_cov_129.672305	IS*1400*	99.01	80.94
FK2020ZBJ035	NODE_10_length_146588_cov_146.778467	IS*26*	100	100
FK2020ZBJ035	NODE_10_length_146588_cov_146.778467	IS*5*	100	99.5
FK2020ZBJ035	NODE_10_length_146588_cov_146.778467	IS*Ec33*	100	99.65
FK2020ZBJ035	NODE_10_length_146588_cov_146.778467	IS*5*	100	99.5
FK2020ZBJ035	NODE_10_length_146588_cov_146.778467	IS*4*	100	88.85
FK2020ZBJ035	NODE_10_length_146588_cov_146.778467	IS*102*	99.91	94.89
FK2020ZBJ035	NODE_10_length_146588_cov_146.778467	IS*1618*	100	90.18
FK2020ZBJ035	NODE_13_length_118936_cov_125.097384	IS*Ehe3*	99.84	91.87
FK2020ZBJ035	NODE_1_length_1288295_cov_96.275921	IS*1400*	87.74	83.66
FK2020ZBJ035	NODE_36_length_3803_cov_315.814010	IS*3000*	100	99.88
FK2020ZBJ035	NODE_39_length_1365_cov_280.046584	IS*5075*	100	99.62
